# Reproducibility of diffusion tensor image analysis along the perivascular space (DTI-ALPS) for evaluating interstitial fluid diffusivity and glymphatic function: CHanges in Alps index on Multiple conditiON acquIsition eXperiment (CHAMONIX) study

**DOI:** 10.1007/s11604-021-01187-5

**Published:** 2021-08-14

**Authors:** Toshiaki Taoka, Rintaro Ito, Rei Nakamichi, Koji Kamagata, Mayuko Sakai, Hisashi Kawai, Toshiki Nakane, Takashi Abe, Kazushige Ichikawa, Junko Kikuta, Shigeki Aoki, Shinji Naganawa

**Affiliations:** 1 Department of Innovative Biomedical Visualization (iBMV), Nagoya University, 65 Tsurumai-cho, Showa-ku, Nagoya, Aichi 466-8550 Japan; 2 Department of Radiology, Nagoya University, Nagoya, Aichi Japan; 3 Department of Radiology, Juntendo University School of Medicine, Tokyo, Japan; 4 Canon Medical Systems Corporation, Otawara, Japan; 5 Department of Radiology, Aichi Medical University, Nagakute, Japan; 6 Department of Radiological Technology, Nagoya University Hospital, Nagoya, Aichi Japan

**Keywords:** Diffusion image, Glymphatic system, DTI-ALPS, Reproducibility, Brain interstitial fluid dynamics

## Abstract

**Purpose:**

The diffusion tensor image analysis along the perivascular space (DTI-ALPS) method was developed to evaluate the brain’s glymphatic function or interstitial fluid dynamics. This study aimed to evaluate the reproducibility of the DTI-ALPS method and the effect of modifications in the imaging method and data evaluation.

**Materials and methods:**

Seven healthy volunteers were enrolled in this study. Image acquisition was performed for this test–retest study using a fixed imaging sequence and modified imaging methods which included the placement of region of interest (ROI), imaging plane, head position, averaging, number of motion-proving gradients, echo time (TE), and a different scanner. The ALPS-index values were evaluated for the change of conditions listed above.

**Results:**

This test–retest study by a fixed imaging sequence showed very high reproducibility (intraclass coefficient = 0.828) for the ALPS-index value. The bilateral ROI placement showed higher reproducibility. The number of averaging and the difference of the scanner did not influence the ALPS-index values. However, modification of the imaging plane and head position impaired reproducibility, and the number of motion-proving gradients affected the ALPS-index value. The ALPS-index values from 12-axis DTI and 3-axis diffusion-weighted image (DWI) showed good correlation (*r* = 0.86). Also, a shorter TE resulted in a larger value of the ALPS-index.

**Conclusion:**

ALPS index was robust under the fixed imaging method even when different scanners were used. ALPS index was influenced by the imaging plane, the number of motion-proving gradient axes, and TE in the imaging sequence. These factors should be uniformed in the planning ALPS method studies. The possibility to develop a 3-axis DWI-ALPS method using three axes of the motion-proving gradient was also suggested.

## Introduction

Since the introduction of the glymphatic system hypothesis by Illif et al. [1], increasing number of studies have attempted to describe the fluid dynamics in the brain parenchyma [2–6]. According to glymphatic system hypothesis, fluid movement in the brain’s interstitium causes the excretion of waste products within the brain. On the other hand, another pathway for waste product removal, the intramural periarterial drainage (iPad) system, has been proposed as the rapid drainage pathway for the interstitial fluid along the basement membrane of smooth muscle cells of the cerebral arteries [7]. Recently, “neurofluids” has been accepted as a collective term for the fluids in which the central nervous system (CNS) is immersed, including the blood, cerebrospinal fluid, and interstitial fluid [8]. This concept helps explore and understand the fluid dynamics within the brain parenchyma. As the impairment of neurofluid dynamics is closely associated with various pathologies, the concept of ‘CNS interstitial fluidopathy’ has also been proposed to indicate the pathologies caused by the abnormal neurofluid dynamics [9].

**Fig. 1:**
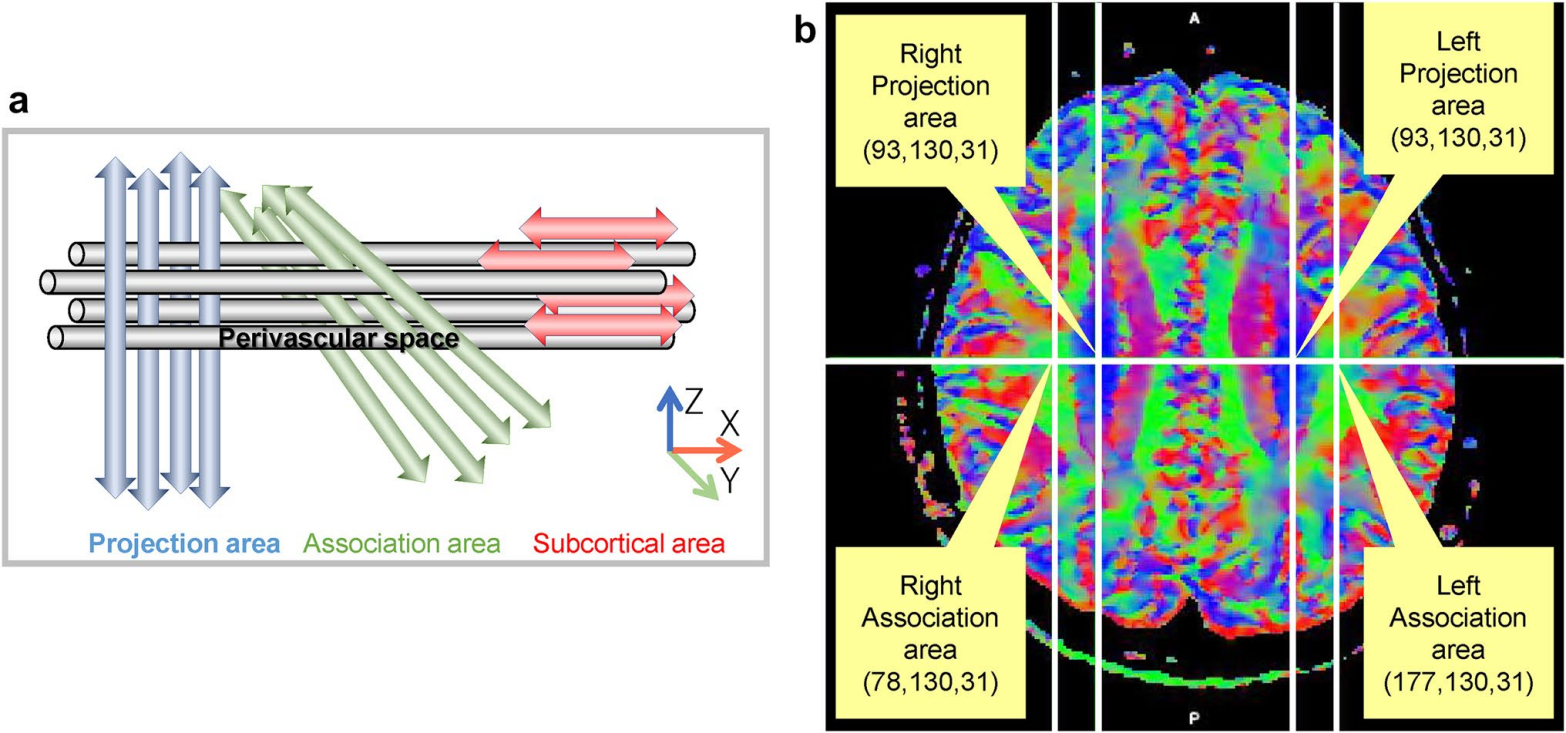
The diffusion tensor image analysis along the perivascular space (DTI-ALPS) method. **a** Schematic illustration of the ALPS index, a schematic illustration of the relationship between the direction of the perivascular space (gray cylinder) and the directions of the fibers in the left hemisphere. Note that the direction of the perivascular space is perpendicular to both projection and association fibers. ALPS index is determined by the following ratio [19]: ALPS-index = mean (Dxxproj, Dxxassoc)/mean (Dyyproj, Dzzassoc). **b** The coordinate for the center of regions of interest (ROIs) regions of interest (ROIs) were placed in the area with the projection fibers (projection area) and association fibers (association area) to measure diffusivity in the three (x, y, and z) directions. The coordinate indicates the center of an ROI, including ‘large sphere,’ ‘small sphere,’ ‘large cube,’ ‘small cube,’ ‘large square,’ and ‘small square’

The diffusion tensor technique is widely used for evaluating the white matter tract in the brain. This non-invasive method evaluates CNS physiology and various pathologies by measuring fractional anisotropy (FA) or apparent diffusion coefficient (ADC) [10–18]. Diffusion tensor image analysis along the perivascular space (DTI-ALPS) is another application of the diffusion tensor method to non-invasively evaluate the interstitial fluid dynamics using diffusion tensor image (DTI) on MRI [19]. The initial study using the DTI-ALPS method observed decreased diffusivity in the direction of the perivascular space in patients with Alzheimer’s disease and mild cognitive impairment, demonstrating the DTI-ALPS method’s effectiveness in evaluating fluid dynamics within the brain parenchyma. The DTI-ALPS method provides ALPS index, which is a ratio of the diffusivity in the direction of the perivascular space and the diffusivity in the direction perpendicular to both major fiber tract and perivascular space, based on a special spatial relationship in the fiber tracts and the perivascular space in the axial imaging plane at the corona radiata [19, 20]. Recently, the DTI-ALPS method has been adopted in several studies evaluating the alteration of the glymphatic system or interstitial fluid dynamics in various pathologies [21–28]. These reports indicated that the ALPS-index is a useful biomarker to evaluate the interstitial fluid dynamics or glymphatic function. However, no study has verified the reproducibility of the DTI-ALPS method and the effect of changes in imaging parameters or calculation methods on the ALPS index. Therefore, the current study (CHanges in Alps index on Multiple conditiON acquIsition eXperiment: CHAMONIX study) evaluated the reproducibility of the DTI-ALPS method and changes in the ALPS index due to different imaging condition or calculation methods (see Fig. 1a).

## Materials and methods

### Subjects

This study was approved by the institutional review board of our institution (2020-0352). The study subjects were seven healthy volunteers, aged 23–51 years (4 females and 3 males, 6 right-handed and 1 left-handed), without any history of neuronal disorder and without any abnormal findings in a conventional MRI brain scan. Written informed consent for the imaging study was obtained from all subjects after the nature of the imaging study had been fully explained.

### Imaging

Images were acquired using two 3 T clinical scanners, namely Vantage Centurian (Canon Medical Systems, Tochigi, Japan) and Magnetom Prisma (Siemens Healthineers, Erlangen, Germany). In this study, Canon Vantage Centurian MRI scanner was mainly used and the standard DTI sequence was determined as follows: echo-planar imaging, repetition time (TR) = 6600 ms, echo time (TE) = 85 ms, diffusion time = 35.7 ms, motion-proving gradient (MPG) = 12 axes, *b* value = 1000 s/mm^2^, anterior-to-posterior (AP) phase encode direction, FOV = 200 mm, matrix = 128 × 128 with interpolation to 256 × 256, slice thickness = 3 mm, slice number = 50, number of averaging = 2, and axial imaging plane on the anterior commissure-posterior commissure (AC-PC) line. Positioning was made manually by the operator. MPGs were aligned to the imaging plane. To apply the TopUp process to correct susceptibility-induced distortions, *b* = 0 images with posterior-to-anterior (PA) phase encode were also acquired. The subjects were centralized to the 16-channel head coil with a neutral position, defined as the head position in which the laser marker light runs through the inferior margin of the orbit and the external auditory meatus. Every measurement was taken between 6 and 8 pm to avoid the circadian rhythm’s effect.

In the current study, the following imaging series were evaluated (Table 1).

1. Test–retest study (data 1a–1d)
  DTI measurements on the standard DTI sequence were repeated four times. Data 1a and 1b were measured sequentially on the same day, and Data 1c and 1d were measured sequentially after 1–5 days.
2. Effect of the imaging plane and head position (data 2a and 2b)
  Modifications were made to the standard DTI sequence (imaging plane on the AC-PC line with a neutral head position) to evaluate the effect of the imaging plane and head position on the DTI-ALPS analysis. Images were acquired on the infra-orbital meatal line’s (IM line) plane with the head in neutral position (data 2a) and images on the AC-PC line with the chin-up head position (data 2b). The IM line was defined as the line drawn between the infra-orbital margin and the external auditory meatus; 7–10° more horizontal to the AC–PC line. The chin-up head position was defined as the head position in which the laser marker light runs through the upper lip and the external auditory meatus. This position comes to approximately 20° chin-up to the neutral position.
3. Effect of averaging and the number of MPG axes (data 3a–3c)
  As the standard DTI sequence was acquired using the 12-axis MPG and averaging = 2, the altered number of averaging and MPG axes were set as follows: 12-axis MPG with averaging = 4 (data 3a), 30-axis MPG with averaging = 1 (data 3b), and 3-axis MPG with averaging = 4 (data 3c). Data 3c corresponds to an orthogonal output of the diffusion-weighted image (DWI).
4. Effect of diffusion time (data 4a and 4b)
  As the standard DTI sequence was acquired with TE = 85 ms (diffusion time = 35.7 ms), the altered diffusion times were set as TE = 100 ms (diffusion time = 40.7 ms; data 4a) and TE = 65 ms (diffusion time = 29 ms; data 4b).
5. Effect of using different scanners (data 5).

**Table 1:** Imaging sequences

Data number	Schedule	Head position	Imaging plane	MPG axes	Average	TE (ms)	Scanner
Test–retest study							
1a	Day 1	Neutral	AC-PC	12	2	85	Centurian
1b	Day 1	Neutral	AC-PC	12	2	85	Centurian
1c	Day 2	Neutral	AC-PC	12	2	85	Centurian
1d	Day 2	Neutral	AC-PC	12	2	85	Centurian
Effect of the imaging plane and head position							
2a	Day 1	Neutral	IM line	12	2	85	Centurian
2b	Day 1	Chin-up	AC-PC	12	2	85	Centurian
Effect of averaging and the number of MPG axes							
3a	Day 2	Neutral	AC-PC	12	4	85	Centurian
3b	Day 2	Neutral	AC-PC	30	1	85	Centurian
3c	Day 2	Neutral	AC-PC	3	4	85	Centurian
Effect of diffusion time							
4a	Day 2	Neutral	AC-PC	12	2	100	Centurian
4b	Day 2	Neutral	AC-PC	12	2	65	Centurian
Effect of using different scanners							
5	Day 2	Neutral	AC-PC	12	2	85	Prisma

*MPG* motion-proving gradients, *TE* echo time, *Neutral* the head position in which the laser marker light runs through the inferior margin of the orbit and the external auditory meatus, *Chin-up* the head position in which the laser marker light runs through the upper lip and the external auditory meatus, *AC-PC* anterior commissure-posterior commissure line, *IM line* infra-orbital meatal line, which is the line drawn from the inferior margin of the orbit to the orifice of the external acoustic meatus

As the standard DTI sequence was acquired using the Canon Vantage Centurian scanner, the altered-scanner images were acquired using the Siemens Magnetom Prisma scanner and identical imaging parameters as follows: echo-planar imaging, TR = 6600 ms, TE = 85 ms, MPG = 12 axes, b value = 1000 s/mm^2^, FOV = 200 mm, matrix = 128 × 128, slice thickness = 3 mm, slice number = 50, number of averaging = 2, and axial imaging plane on the AC–PC line.

### Image processing

Data obtained using DTI, in which the number of MPG was 12 axes and 30 axes (i.e., other than Data 3c), were processed using FMRIB Software Library version 6.0 (FSL; Oxford Centre for Functional MRI of the Brain, Oxford, UK; www.fmrib.ox.ac.uk/fsl) [29]. Diffusivity maps of each subject were obtained in the direction of the x-axis (right-left; Dxx), y-axis (anterior–posterior; Dyy), and z-axis (inferior-superior; Dzz) [26]. The FA map of all individuals was then converted into FMRIB58_FA standard space using both linear and nonlinear transformations.

One subject with the smallest degree of warping was selected for the region of interest (ROI) placement. Using this subject’s color-coded FA map, ROIs were placed in the projection and association areas at the level of the lateral ventricle body (Fig. 1b). The size and shape of the ROI of six different patterns with matched center positions were applied. The standard ROI in the current study was a large spherical ROI {diameter, 12 pixels [≈ 9.4 mm]}. The variations were small spherical ROI {diameter, 8 pixels [≈ 6.3 mm]}, large cubical ROI (each side, 12 pixels), small cubical ROI (each side, 8 pixels), large square ROI (each side, 12 pixels), and small square ROI (each side, 8 pixels).

Data 3c was obtained using the 3-axis MPG (x, y, and z), which the FMRIB Software Library could not process; thus, processed using ImageJ 1.50b (U. S. National Institutes of Health, Bethesda, Maryland, USA, https://imagej.nih.gov/ij/) [30]. Diffusivity maps with x, y, and z directions were generated, and composite images with channels of red (diffusivity of the x-direction), green (diffusivity of the y-direction), and blue (diffusivity of the z-direction) were composed. Circular ROIs (diameter, 12 pixels) were placed in the projection and association areas at the level of the left lateral ventricle body, and diffusivity of the x, y, and z directions were measured.

On the ROIs, the x-, y-, and z-axis diffusivity were measured, and the ALPS index was calculated for each case, which is a ratio of the mean of the x-axis diffusivity in the projection area (Dxxproj) and the x-axis diffusivity in the association area (Dxxassoc) to the mean of the y-axis diffusivity in the projection area (Dyyproj) and the z-axis diffusivity in the association area (Dzzaccoc):

$${\text{ALPS index}}\, = \,\left( {{\text{mean }}\left( {{\text{Dxxproj}},{\text{Dxxassoc}}} \right)} \right)/({\text{mean }}({\text{Dyyproj}},{\text{Dzzassoc}})).$$

The ALPS index of the right-side projection area and association area (ALPS-index-R), the left-side projection area and association area (ALPS-index-L), and the average of the bilateral ALPS index (ALPS-index-Bil) were calculated.

### Statistical analysis

Statistical analyses were performed using R version 4.0.2 software (R Foundation for Statistical Computing, Vienna, Austria. URL https://www.R-project.org/.) to evaluate the reproducibility of the ALPS-index measurement and the effect of the imaging plane, head position, imaging sequence, or difference of the scanner on the ALPS index. A *p* value of less than 0.05 was considered statistically significant.

1. Test–retest study (data 1a–1d)
  Intraclass correlation coefficient (ICC) was measured to determine the ALPS-index measurement’s reproducibility. The ALPS-index measurements from the four datasets using standard DTI sequence with identical positioning and imaging sequence were analyzed. ICCs were calculated for the ALPS indices for different ROI shapes and sizes. Additionally, ICCs were calculated for ALPS-index-R, ALPS-index-L, and ALPS-index-Bil.
2. Effect of the imaging plane and head position (data 2a and 2b)
  A paired *t* test was performed to evaluate the difference in the imaging plane by comparing the mean ALPS-index-Bil using large spherical ROI (data 1a–1d; AC-PC line/neutral position) and data 2a (IM line/neutral position). Similarly, a paired t test was performed to evaluate the difference in the head position by comparing the mean values of data 1a–1d (AC-PC line/neutral position) and data 2b (AC–PC line/chin-up position). To evaluate ALPS-index-Bil’s reproducibility and correlation between different head positions or imaging planes, ICCs and Pearson’s correlation coefficients were calculated.
3. Effect of averaging and the number of MPG axes (data 3a–3c)
  The effect of averaging on the ALPS-index-Bil value was evaluated by performing a paired *t* test to compare the mean value of the standard DTI sequence (data 1a–1d) acquired with two-time averaging, and data 3a, acquired with four-time averaging. ICCs and Pearson’s correlation coefficients were also calculated.
  The effect of the number of MPG axes on the ALPS-index-Bil value using large spherical ROI was evaluated by performing a paired *t* test to compare the mean values of the standard DTI sequence acquired using 12-axis (data 1a–1d) and 30-axis MPG (data 3b). ICCs and Pearson’s correlation coefficients were calculated to evaluate the reproducibility and correlation between ALPS-index-Bil and the number of MPG axes.
  A paired *t* test was performed to compare the ALPS-index-Bil values for the images with a 3-axis MPG (i.e., DWI; data 3c) and the standard DTI sequence’s mean value (data 1a–1d). ICCs and Pearson’s correlation coefficients were calculated to evaluate ALPS-index-Bil value’s reproducibility and correlation between DTI and DWI measurements.
4. Effect of diffusion time (data 4a and 4b)
  The effect of diffusion time on ALPS-index-Bil at various TEs, including 100 ms (data 4a), 85 ms (data 1a–1d), and 65 ms (data 4b) using large spherical ROI were compared using two-way ANOVA with repeated measures.
5. Effect of using different scanners (data 5)

A paired *t* test was performed to evaluate the effect of scanners by comparing the mean ALPS-index-Bil values acquired using large spherical ROI, identical imaging parameters as the standard DTI sequence, and Magnetom Prisma (data 5), and the value acquired with the standard DTI sequence and using a Vantage Centurian scanner on the same day (data 1c and 1d). ICCs and Pearson’s correlation coefficients were calculated to evaluate the reproducibility and correlation between ALPS-index-Bil values obtained using different scanners.

## Results

**Fig. 2:**
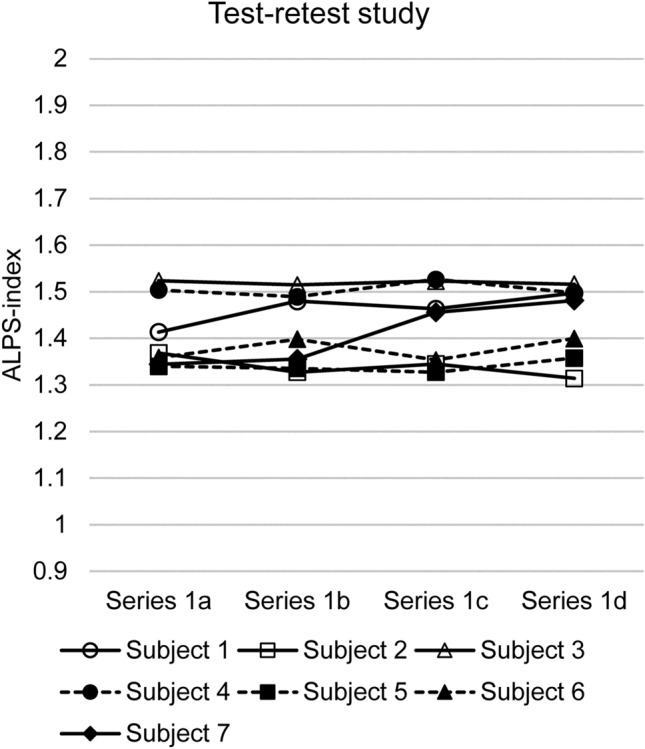
The result of the test–retest study using the standard diffusion tensor image (DTI) sequence. The results of the test–retest study for evaluating the bilateral ALPS index using the standard DTI sequence and large spherical region of interest for four measurements in each of the seven study subjects. Data 1a and 1b were measured sequentially on the same day, and Data 1c and 1d were measured sequentially on a different day. The intraclass correlation coefficient for these measurements was 0.828, which means almost perfect agreement

**Fig. 3:**
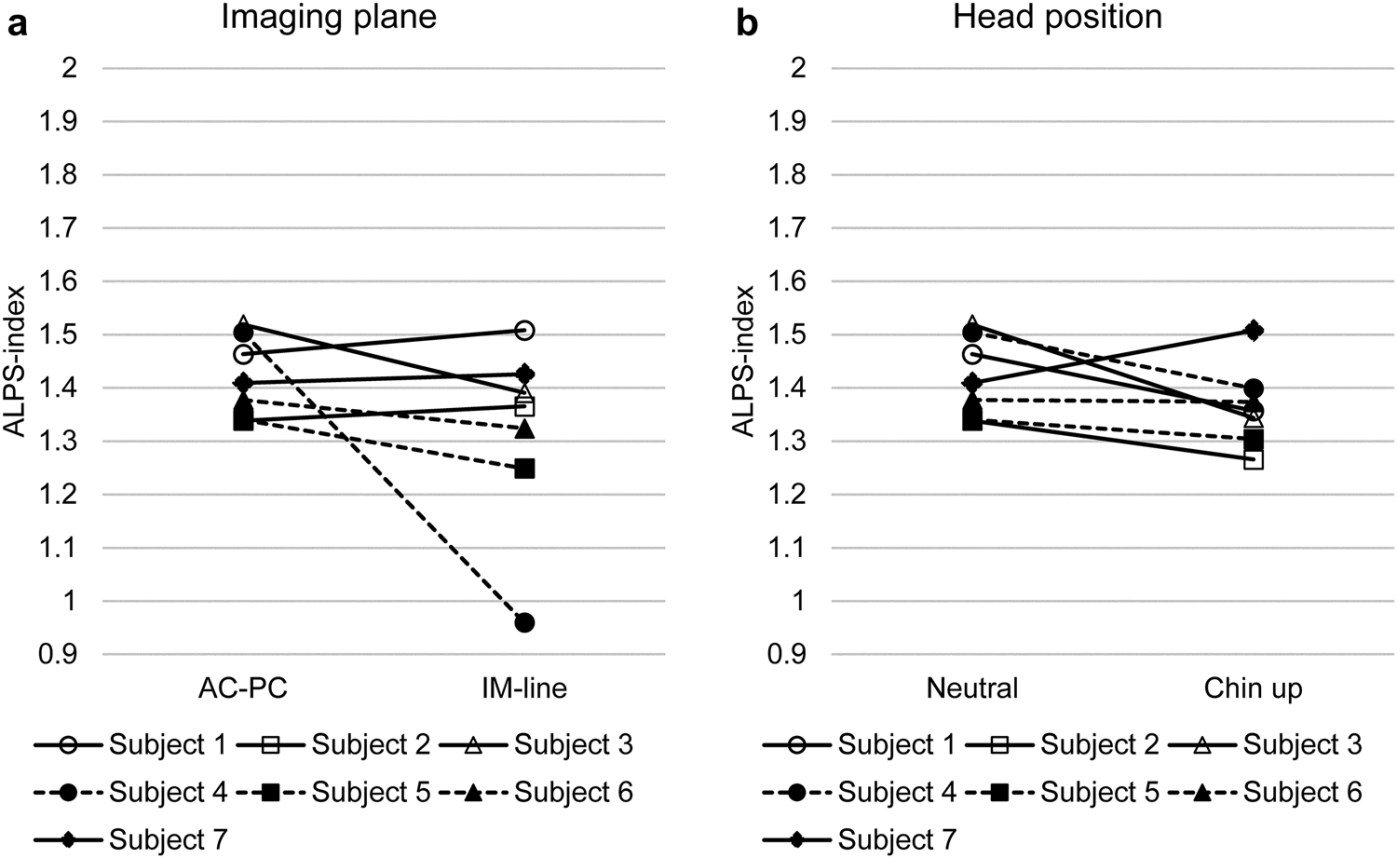
The effect of imaging plane and head position. **a** Effect of the imaging plane. The values of bilateral ALPS index with a large spherical region of interest calculated from the images on the anterior commissure-posterior commissure line and infra-orbital meatal line are shown. The intraclass correlation coefficient for the bilateral ALPS-index value was 0.19, indicating low-level reproducibility. **b** Effect of head position. The values of bilateral ALPS index with a large spherical region of interest calculated from the images on the anterior commissure-posterior commissure line acquired with a neutral head position and a chin-raising (approximately 20°) position. The intraclass correlation coefficient for the bilateral ALPS-index value was 0.22, indicating low-level reproducibility

**Fig. 4:**
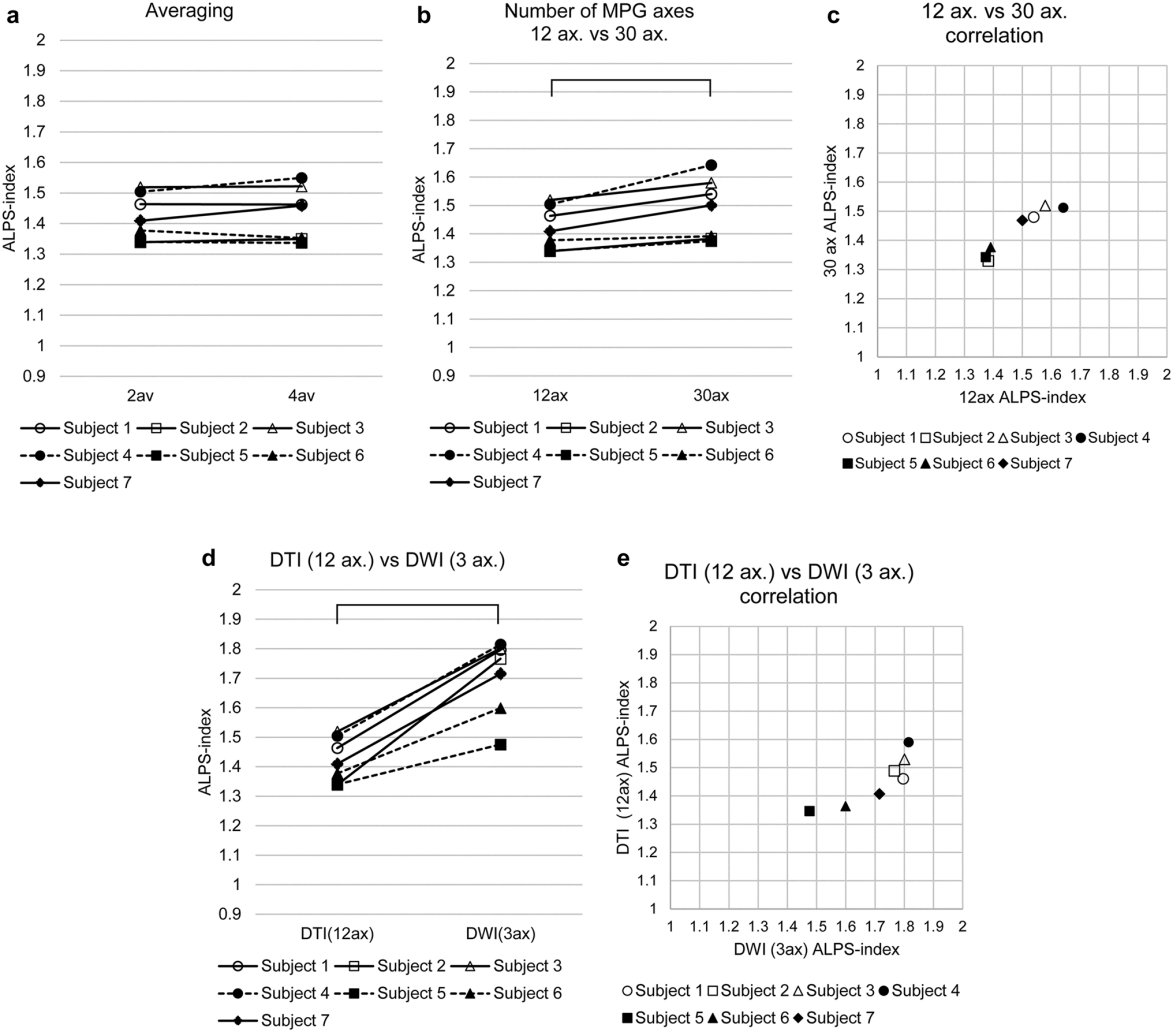
The effect of averaging and the number of motion-proving gradient (MPG) axes **a** Effect of the number of averaging. The effect of averaging the bilateral ALPS-index value using a large spherical region of interest is shown. The intraclass correlation coefficient for the bilateral ALPS-index value was 0.96, indicating very high-level reproducibility. **b** Effect of the number of MPG axes (12 axes versus 30 axes). The paired *t* test showed a statistically significant difference (*p* = 0.0056) between 12 and 30 axes. **c** Effect of the number of MPG axes (12 axes versus 30 axes): analysis for the correlation. The correlation coefficient between the bilateral ALPS-index values at 12 axes and 30 axes was as high as 0.97. **d** Effect of the number of MPG axes (12 axes versus 3 axes): analysis for the difference. Paired *t* test showed a statistically significant difference (*p* = 0 0.000058) between 12 and 3 axes. **e** Effect of the number of MPG between 12 and 3 axes: analysis for the correlation. The correlation coefficient between the bilateral ALPS-index values at 12 axes and 3 axes was as high as 0.86

**Fig. 5:**
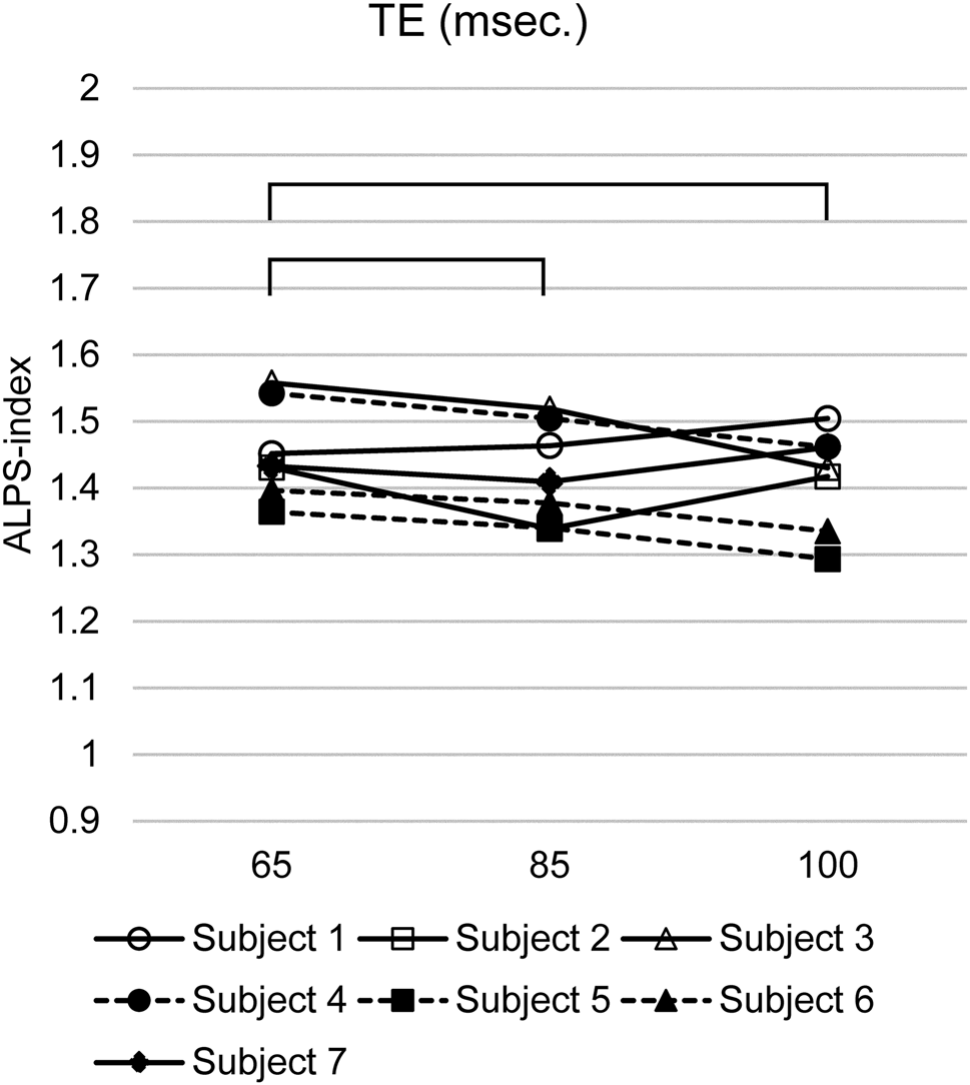
Effect of diffusion time. The bilateral ALPS index from the images with TE = 65 ms showed a significantly (*p* < 0.05) larger value than the images with TE = 85 ms and TE = 100 ms

1. Test–retest study (data 1a–1d)
  The results of the test–retest study for the ALPS-index-Bil using a large spherical ROI for data 1a–1d are shown in Fig. 2. The reproducibility evaluated using the ICC is shown in Table 2, in which the ICC for the ALPS-index-R, ALPS-index-L, and ALPS-index-Bil are shown for each combination of the size and shape of ROIs.
  The ICC values ranged from 0.542 to 0.932, indicating a ‘substantial’ or ‘almost perfect’ correlation [31] throughout the laterality of the calculation (right, left, or bilateral) and ROI sizes and shapes. The ICC values were higher when a large ROI was applied and stable when the bilateral ALPS-index’s averaging was done.
2. Effect of the imaging plane and head position (data 2a and 2b)
  Figure 3 shows the effect of the imaging plane (Fig. 3a) and head position (Fig. 3b) on the ALPS-index-Bil using a large spherical ROI. The paired *t* test showed no significant difference (*p* = 0.23) between the ALPS-index-Bil evaluated using the AC-PC (data 1a–1d) and IM lines (data 2a). Furthermore, the ICC for the ALPS-index-Bil values was 0.19, indicating low-level reproducibility; the Pearson’s correlation coefficient was 0.18.
  There was no significant difference (*p* = 0.14) between the ALPS-index-Bil values evaluated in neutral (data 1a–1d) and chin-up (data 2b) positions. The ICC for the ALPS-index-Bil values was 0.22, indicating low-level reproducibility; the Pearson’s correlation coefficient was 0.33.
3. Effect of averaging and the number of MPG axes (data 3a–3c)
  The paired *t* test showed no significant difference (*p* = 0.31) between the ALPS-index-Bil values calculated by two-time averaging (data 1a–1d) and four-time averaging (data 3a) (Fig. 4a). The ICC for the ALPS-index-Bil values was 0.96, indicating very high-level reproducibility; the Pearson’s correlation coefficient was 0.98 (*p* < 0001).
  The effect of the number of MPG axes (12 axes and 30 axes; data 1a–1d and data 3b) on the ALPS-index-Bil is shown in Fig. 4b. The paired *t* test showed a statistically significant difference (*p* = 0.0056) between 12-axis and 30-axis MPGs. The ICC for the ALPS-index-Bil values was 0.68. The correlation coefficient between 12-axis (data 1a–1d) and 30-axis (data 3b) MPGs’ ALPS-index-Bil values was 0.97 (*p* < 0001) (Fig. 4c).
  The ALPS-index-Bil value obtained using a 3-axis MPG (data 3c), which is the orthogonal output of DWI, was compared with the mean value of the standard DTI sequence (data 1a–1d), which was acquired using the 12-axis MPG (Fig. 4d). The paired *t* test showed a statistically significant difference (*p* = 0 0.000058) between 12-axis and 3-axis MPG-based ALPS-index-Bil values. Although the ICC for the ALPS-index-Bil values was as low as 0.22, the correlation coefficient between the ALPS-index-Bil values evaluated using 12-axis and 3-axis MPGs was 0.86 (*p* < 0.05) (Fig. 4e).
4. Effect of diffusion time (data 4a and 4b)
  The effect of diffusion time at various TEs (66, 85, 100 ms) using a large spherical ROI is shown in Fig. 5. The two-way ANOVA with repeated measures showed a statistically significant difference (*p* = 0.030) between the ALPS-index-Bil values evaluated using these three TEs. The ALPS-index-Bil value for TE = 65 ms (data 4b) showed a significantly larger value (*p* < 0.05) than the values obtained for TE = 85 ms (data 1a–1d), and TE = 100 ms (data 4a).
5. Effect of using different scanners (data 5).

**Table 2:** Intraclass correlation coefficient (ICC) for the test–retest study

	ALPS-index-Bil	ALPS-index-R	ALPS-index-L
Large sphere	0.828	0.812	0.816
Large cube	0.886	0.716	0.932
Large square	0.877	0.809	0.901
Small sphere	0.781	0.883	0.542
Small cube	0.786	0.814	0.678
Small square	0.775	0.842	0.644

*ALPS* analysis along the perivascular space, *ALPS-index-Bil* bilateral ALPS index, *ALPS-index-R* ALPS index of the right-side projection area and association area, *ALPS-index-L* ALPS index of the left-side projection area and association area

**Fig. 6:**
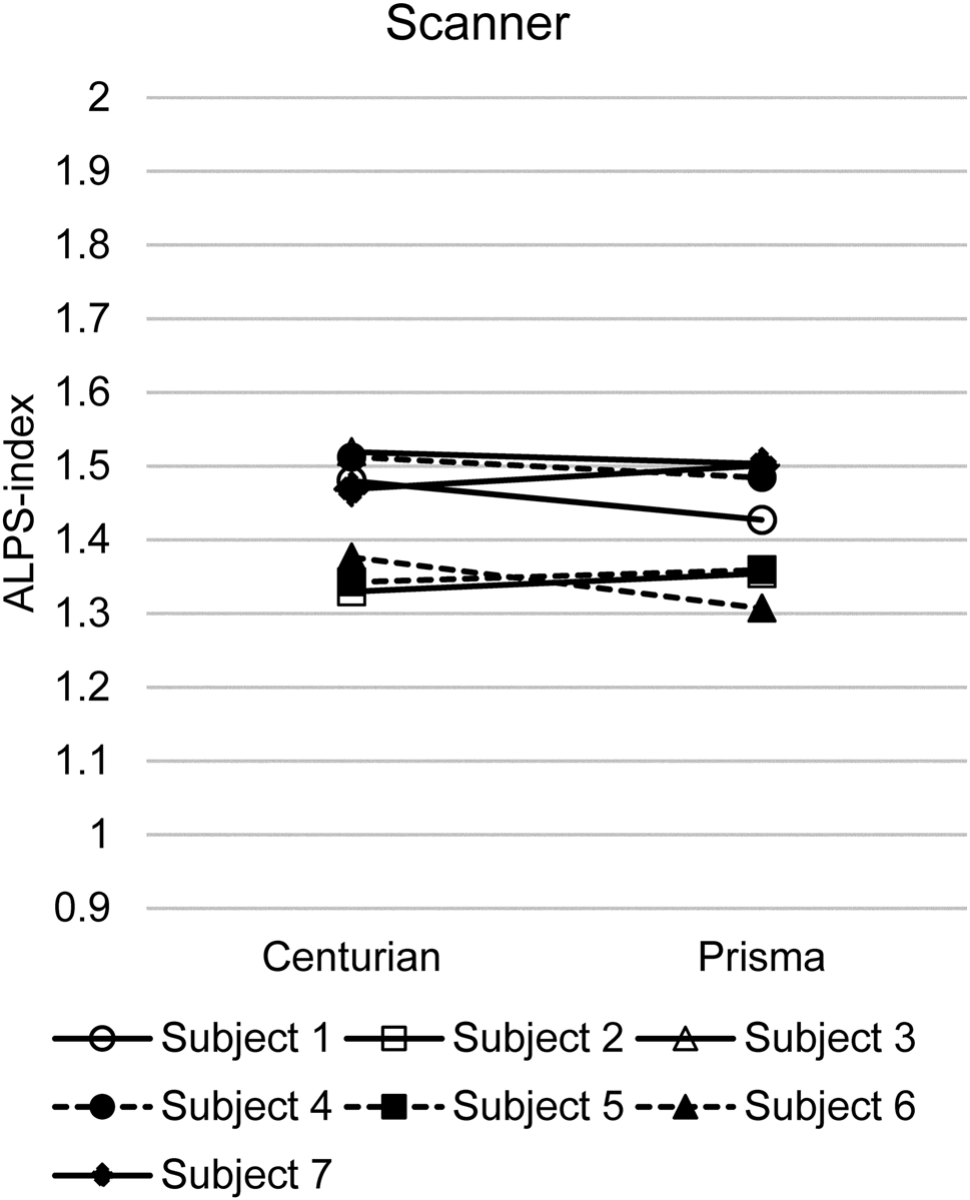
Effect of the scanner. The paired *t* test showed no significant difference (*p* = 0.70) between bilateral ALPS-index values obtained using Vantage Centurian (Canon medical systems) and Magnetom Prisma (Siemens Healthineers)

Different scanners were compared using identical scanning parameters (Fig. 6). The paired *t* test showed no significant difference (*p* = 0.70) between the Vantage Centurian (Canon medical systems) and Magnetom Prisma (Siemens Healthineers) scanners. The ICC for the ALPS-index-Bil values was 0.87, indicating very high-level reproducibility; Pearson’s correlation coefficient was 0.85 (*p* < 0.05).

## Discussion

Since the glymphatic system hypothesis was introduced, several imaging methods have been proposed to evaluate the glymphatic system’s function or interstitial fluid dynamics. A tracer study is one of the most efficient methods to visualize the interstitial fluid dynamics. The initial report on the glymphatic system hypothesis used a fluorescent tracer and a laser-scanning microscope [1]. After the initial report, the follow-up tracer studies used the intrathecal administration of gadolinium-based contrast agent (GBCA) on MRI [32, 33]. While the fluorescent tracer and laser-scanning microscope can observe only the surface of the brain, the MRI provides a tomographic image and allows evaluation of the whole brain. Several institutions use intrathecal administrations of GBCA in human subjects for the clinical diagnosis and have carefully evaluated the procedure and appropriate dose decisions and received approval of their institutional certification boards [34, 35]. However, the intrathecal administration of GBCA in humans has not been approved in any country [36]. In this context, several trials have evaluated the glymphatic function through the intravenous administration of GBCA [37–41]. In contrast to the tracer study, including intravenous GBCA, provides information about the tracer’s movement as the integral of the elapsed time, and the diffusion method provides information about the tissue water molecules at the moment the MPGs are applied. Therefore, the diffusion-based techniques may provide very different information compared to the tracer studies. Another advantage of the diffusion method is that it is non-invasive and can be used repeatedly, which is important point for clinical applications. The DTI-ALPS method is one of the approaches to evaluate the glymphatic function or interstitial fluid dynamics by observing water diffusion in the direction of the perivascular space.

The DTI-ALPS method is used for evaluating the glymphatic system’s function or the interstitial fluid dynamics, and this method has been adopted in several studies evaluating the alteration of the glymphatic system or interstitial fluid dynamics in various pathologies [21–28]. One of the report showed that results of ALPS method were significantly related to glymphatic clearance function calculated on MRI by intrathecal administration of GBCA [28]. The current study evaluated the reproducibility or robustness of the DTI-ALPS method for the fixed imaging sequence and the effect of modifying the method of image acquisition and data evaluation. Our test–retest study showed reproducibility, indicating that the DTI-ALPS method provides robust results as far as fixing the imaging method, imaging plane, head position, and evaluation method is concerned. In the original report on DTI-ALPS, the ALPS index was measured only in the dominant hemisphere. However, the result of the study on the variation for ALPS-index calculation shown in Table 2 indicates that the ALPS index with the averaging of bilateral index showed a higher yield of ICC compared to the unilateral calculation. The larger size of the ROIs {diameter, 12 pixels [≈9.4 mm]} also showed a higher yield than the smaller ROIs. This larger size matches the size of the projection fiber area and association fiber area on the imaging plane at the level of the lateral ventricle body; therefore, the large-size ROI may benefit by canceling the spatial inhomogeneity within the brain parenchyma. The shape of the ROIs (square, cube, or sphere) did not bring large differences compared to the size of the ROIs.

In contrast to the robustness of the ALPS index calculated using the fixed imaging method, some modifications in the imaging method altered the ALPS-index values. Most of all, alteration in the imaging plane and head position largely influenced the reproducibility of the ALPS index. Data 2b showed that the ALPS index varies by head position with 20°. In addition, the difference of the imaging plane to the IM line, which was 7–10°, brought more severely impaired reproducibility, especially subject 4 showed a very large difference. We checked the original image of this subject and found that the superior longitudinal fiber (SLF: association fiber) was shorter than the other subjects, and the automated ROI was placed outside of SLF. These phenomena may be partially due to the actual angle of the projection fiber or association fiber does not completely rectangle with the direction of standard coordinates in the individual subjects. We also find the lower reproducibility in the ALPS-index when chin-up position was made. The reason for the difference is not clear, but some alteration in CSF dynamics might occur in such head position. We studied the difference in the number of averaging, because a previous report indicated that the higher noise increases the FA value [42]. However, the number of averaging did not alter the ALPS-index values in our study, indicating that the difference in signal-to-noise ratio does not affect the robustness of the ALPS index. It was interesting that there was a large difference due to the number of MPG axes in the current study. The ALPS-index values were larger for the 30-axis MPG than the 12-axis MPG. Some reports have indicated that the higher number of MPG axes reduces the FA value [43, 44] whereas another report suggested that the higher number of MPG axes increases the FA value [45]. Nevertheless, alteration in the shape of the diffusion ellipsoid is brought by the difference in the number of MPG axes, leading to the alteration of the ALPS-index values. A comparison between the 12-axis DTI and 3-axis DWI also showed a large difference in the ALPS-index values. Also, the ALPS-index values for DTI and DWI showed good correlation. The shorter diffusion time in the sequence with a short TE resulted in a larger ALPS index in the current study. Reports have suggested that the ADC or FA value is altered by changing the TE [46, 47]. Similar to the number of MPG axes, diffusion time also alters the shape of the diffusion ellipsoid and leads to the alteration of the ALPS index. There is limited reproducibility for the quantitative values from the diffusion technique, including ADC or FA values, when imaging parameters or calculation methods are modified [48–50]. The robustness of the quantification when the imaging conditions are fixed has been previously reported [51, 52]. Our comparison of scanners proved that the ALPS index is robust to the differences in the scanners as long as identical imaging parameters and imaging planes are used. Although we made comparison between Canon and Siemens scanners in the current study, similar results would be expected with GE and Philips scanners. According to the above-mentioned observation, requirement for the imaging sequence itself does not seem to be strict for DTI-ALPS method. In this study, 12-axis MPG and 85 ms TE could provide sufficient reproducibility. However, the important point is the uniformity of the imaging sequence, including the head position or imaging plane. The comparison of the ALPS-index provided by different imaging sequences does not make sense.

In the current study, we tried ALPS measurement using 3-axis MPG or the DWI-ALPS method. Our comparison of 12-axis DTI and 3-axis DWI, showed significantly different ALPS-index values. However, the standard DTI-ALPS method and 3-axis MPG ALPS-index values showed a very high correlation. This suggests that although the values from the DTI-ALPS and DWI-ALPS methods cannot be discussed on the same scale, the latter can substitute the former for comparing the values obtained by the same method. DWI is more widely used in the clinical imaging practice than DTI, which takes a long acquisition time. If the DWI-ALPS evaluation method gets established, the glymphatic function or interstitial fluid dynamics would be evaluated simultaneously in the daily clinical images.

Our study has some limitations. First, the sample size is very small. However, we believe that our study elucidated the essential information regarding the DTI-ALPS method. Another limitation is that the standard DTI sequence used the 12-axis MPG. Our analysis showed that the number of MPG axes is important for the ALPS-index value; it was unclear at the time of planning the study. We did not evaluate the effect of the resolution in this study. A report has suggested that the smaller voxel side increases FA value in DTI analysis [53]. Therefore, the resolution should be considered while planning DTI-ALPS studies. We set *b* value of MPG as 1000 s/mm^2^ in the current study which has not proven to be optimal for ALPS method. Another series of study is planned for evaluating optimal b value for ALPS method.

In conclusion, the ALPS-index values obtained using the DTI-ALPS method to evaluate glymphatic function or interstitial fluid dynamics are robust under unified imaging method and post-processing method even when different scanners are used. However, ALPS-index is influenced by the imaging plane, number of MPG axes and TE in the imaging sequence, and these factors should be standardized for studying the ALPS method. This finding is similar to other quantifications using a diffusion method. Furthermore, our results suggest the possibility to develop a DWI-ALPS method which uses 3 axes of MPG and can be acquired simultaneously with the daily clinical practice.
